# Evaluation of Left Ventricular Systolic Function in Patients with Coronary Microvascular Dysfunction by Three-Dimensional Speckle-Tracking Imaging

**DOI:** 10.21470/1678-9741-2020-0455

**Published:** 2022

**Authors:** Yu Ziheng, Hongfen Pan, Zhenfeng Cheng, Kongjie Lu, Huanhuan Hu

**Affiliations:** 1 Department of Cardiology, Huzhou Central Hospital, Affiliated Central Hospital of Huzhou University, Huzhou, People’s Republic of China.; 2 Department of Nephrology, Huzhou Fourth Hospital, Huzhou, People’s Republic of China.

**Keywords:** Heart Ventricles. Coronary Angiography. Coronary Stenosis. Echocardiography, Three-Dimensional. Ventricular Function, Left

## Abstract

**Introduction:**

The objective of this study is to evaluate the left ventricular systolic function of patients with coronary microvascular dysfunction (CMD) using the three-dimensional speckle-tracking imaging (3D-STI) technique.

**Methods:**

From June 2018 to June 2019,72 subjects from Huzhou Central Hospital were enrolled, including 42 CMD in-patients with typical chest pain or chest tightness and positive treadmill exercise stress test, but without coronary stenosis on coronary angiography, (the CMD group) and another 30 healthy individuals who were undergoing physical examinations in an outpatient clinic (the control group). Using 3D-STI technique, the global longitudinal strain (GLS), global radial strain (GRS), global circumferential strain (GCS), global area strain (GAS), and left ventricle were measured.

**Results:**

Compared with the control group, GLS and GAS were significantly reduced in the CMD group (*P*<0.05), while GRS and GCS were similar in both groups (*P*>0.05). Univariate logistic regression analysis showed that GLS and GAS were the influencing factors of CMD. For the diagnosis of CMD, the area under the receiver operating characteristic (ROC) curve of GLS was 0.883, and the area under the ROC curve of GAS was 0.875. GAS of -29.3% (log-rank test chi-square=34.245, *P*<0.001) was a strong predictor of major adverse cardiac events.

**Conclusion:**

3D-STI technique has obvious advantages in the evaluation of the left ventricular systolic function for CMD patients. Moreover, 3D-STI parameters, especially GLS and GAS, can detect the early abnormal changes in the ischaemic myocardium. Being timelier and more sensitive than echocardiography, 3D-STI should be recommended for clinical application.

**Table:** 

Abbreviations, acronyms & symbols				
3D-STI	= Three-dimensional speckle-tracking imaging		GAS	= Global area strain
A	= Left atrioventricular valve late diastolic blood flow peak		GCS	= Global circumferential strain
B	=Regression coefficient		GLS	= Global longitudinal strain
CI	= Confidence interval		GRS	= Global radial strain
CMD	= Coronary microvascular dysfunction		LVEDd	= Left ventricular end-diastolic diameter
CTA	= Computed tomography angiography		LVEF	= Left ventricular ejection fraction
Df	= Degree of freedom		LVESd	= Left ventricular end-systolic diameter
E	= Left atrioventricular valve early diastolic blood flow peak		MACE	= Major adverse cardiac events
E '	= Left atrioventricular annulus diastolic early motion velocity		OR	= Odds ratio
ED	= End-diastolic		ROC	= Receiver operating characteristic
ES	= End-systolic		SE	= Standard error

## INTRODUCTION

Coronary microvascular dysfunction (CMD) refers to a group of clinical syndromes with typical exertional angina, positive electrocardiogram or stress test results, and normal coronary angiography, for which the coronary artery spasm can be excluded. CMD is also known as microvascular angina due to the widespread presence of coronary microvascular endothelial dysfunction ^[1]^. The pathophysiological mechanism of CMD is still unclear. Although CMD has a good long-term prognosis, patients with chest pain have a relatively high mortality ^[2]^. In addition, patients with CMD may present with ST segment depression on an electrocardiogram, but have normal or almost normal coronary arteries on angiography ^[3]^. This study aimed to use three-dimensional speckle-tracking imaging (3D-STI) technique to evaluate the left ventricular systolic function of CMD patients for early detecting the ischaemic myocardium and judging patients’ prognosis.

## METHODS

### Subjects

In the CMD group, 42 CMD in-patients with typical chest pain or chest tightness and positive treadmill exercise stress test, without coronary stenosis on coronary angiography, were selected from the Department of Cardiology, Huzhou Central Hospital, between June 2018 and June 2019. None of them received treatment with antianginal drugs. Meanwhile, 30 healthy subjects undergoing physical examination in the outpatient clinic during the same period were selected as the control group. All subjects had negative results after coronary angiography or scanning by 128-row, high-resolution, coronary spiral computed tomography angiography (CTA). All women in this study were not menopausal or had stable hormone levels after hormone replacement therapy for menopause. No subject was in menstruating stage. This study was approved by the Ethics Committee of Huzhou Central Hospital (20200206-01). All subjects were informed of the study details and signed a consent form.

### Inclusion and Exclusion Criteria

For the CMD group, the inclusion criteria were as follows: i) aged 25-75 years; ii) typical fatigue angina (with or without resting angina and dyspnoea); iii) positive exercise treadmill test (cardiologist-reported ischemic changes or abnormal ST results defined as an up-sloping ST change ≥ 2 mm or downsloping or horizontal ST change ≥ 1 mm) or positive myocardial load perfusion imaging; iv) the echocardiography, coronary angiography, or CTA showed that the degree of coronary artery stenosis was < 50%, and there was no ST elevation or angina occurrence at night. The exclusion criteria were as follows: uncontrolled hypertension or diabetes mellitus, left ventricular hypertrophy, cardiomyopathy, coronary artery spasm, valve disease, severe liver or kidney dysfunction, malignant tumour, autoimmune disease, or drug addiction.

For the control group, the inclusion criteria were as follows: i) aged 25-75 years; ii) no clinical indication. The exclusion criteria were the same for those in the CMD group.

### Conventional Echocardiography

Cardiac colour ultrasound (GE vivid E9 colour Doppler echocardiographer; M5S probe, 4V volume probe, frequency 1.7-3.3mHz) was used to measure the left ventricular end-diastolic diameter (LVEDd), left ventricular end-systolic diameter (LVESd), left atrioventricular valve early diastolic blood flow peak/left atrioventricular valve late diastolic blood flow peak (E/A), and E/left atrioventricular annulus diastolic early motion velocity (E'); the left ventricular ejection fraction (LVEF) was calculated using the biplanar Simpson’s method.

### 3D-STI Technique

**Fig. 1:**
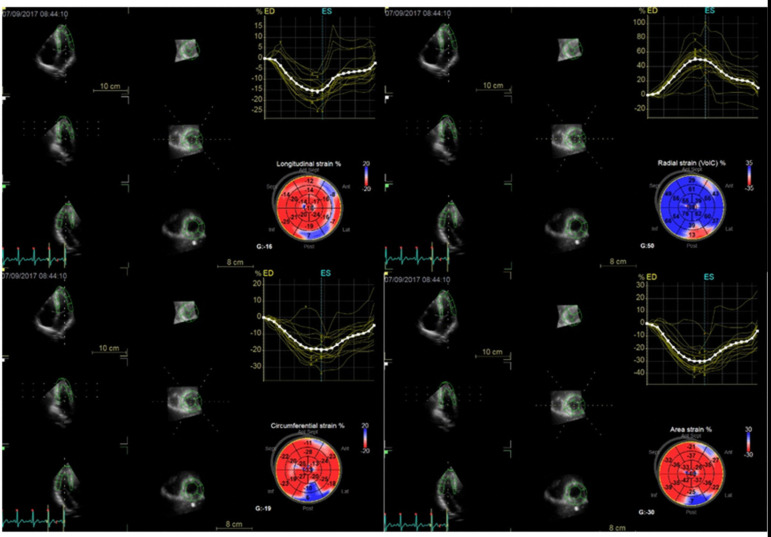
Three-dimensional speckle-tracking imaging of the left ventricle. EchoPAC software was used to trace the left ventricular endocardium and epicardial edge to obtain various three-dimensional strain indicators. ED=end-diastolic; ES=end-systolic

3D-STI was performed using the GE vivid E9 colour Doppler echocardiographer with a matrix-array transducer (M5S) (there was no obvious difference in obtaining 3D strain values using equipment from different inter vendors [*e.g*., Philips], so the GE vivid series were selected in this study). Before acquisition, echocardiographic images were optimized for visualization of the endocardial border. The image acquisition was performed in the apical four-chamber view. Using GE EchoPAC software, a semiautomated and border-tracking analysis was performed. The left ventricle was automatically divided into 16 segments by the software, and the global longitudinal strain (GLS), global radial strain (GRS), global circumferential strain (GCS), and global area strain (GAS) of the endocardium and epicardium in the 16 segments were measured (Figure 1).

### Follow-Up and Major Adverse Cardiac Events

Follow-up was performed for 24 weeks. Major adverse cardiac events (MACE) included recurrent angina, acute myocardial infraction, heart failure, and coronary revascularization. During the follow-up period, when MACE occurred, the patients were considered to have endpoint events. The follow-up was discontinued, and antianginal drugs were given at that time.

### Statistical Analysis

The statistical analysis was performed using the IBM Corp. Released 2013, IBM SPSS Statistics for Windows, Version 22.0, Armonk, NY: IBM Corp. software, and the measurement data were expressed as mean±standard deviation. A *t*-test was used for comparison between two groups. The comparison of numeration data was performed using the χ^2^ test. Univariate logistic regression analysis was used for single-factor analysis. The specificity and sensitivity were determined by calculating the area under the receiver operating characteristic (ROC) curve. A Kaplan-Meier curve was used for survival prognosis analysis. The Bland-Altman repeatability test was used for intraobserver and interobserver consistency (both the interobserver variability and intraobserver variability were < 15%). *P*<0.05 was considered statistically significant.

## RESULTS

### Comparison of Baseline Data Between the Groups

The gender, age, body mass index, systolic blood pressure, diastolic blood pressure, fasting blood glucose, and low-density lipoprotein levels in control and CMD groups were compared. Each index had no significant difference between the groups (*P*>0.05) (Table 1).

**Table 1:** Comparison of baseline data between the groups.

	Control group	CMD group	t/χ^2^	P-value
n	30	42		
Age (years)	49.43±9.76	47.45±12.34	0.730	0.468
Male, n (%)	15(50.00)	23(54.76)	1.290	0.256
Body mass index (kg/m^2^)	22.54±3.15	23.41±1.84	1.474	0.145
Fasting blood glucose (mmol/L)	5.71±1.53	5.60±1.08	0.358	0.722
Low-density lipoprotein (mmol/L)	2.72±0.69	2.69±0.47	0.220	0.827
Systolic pressure (mmHg)	123.80±9.19	126.55±10.62	1.144	0.256
Diastolic pressure (mmHg)	69.93±10.62	68.64±6.90	0.625	0.534

CMD=coronary microvascular dysfunction

Comparison of Conventional Echocardiography Parameters Between the Groups

Comparison of the cardiac colour ultrasound indexes between control and CMD groups showed that the LVEDd, LVESd, E/A, E/E', and LVEF values had no significant difference between the groups, respectively (*P*>0.05) (Table 2).

**Table 2:** Comparison of parameters detected by colour Doppler echocardiography between the groups.

	Control group	CMD group	t	*P*-value
n	30	42		
LVEDd (mm)	44.96±3.42	45.86±2.19	1.361	0.178
LVESd (mm)	25.82±2.88	26.06±3.19	0.328	0.744
E/A	1.41±0.18	1.37±0.25	0.748	0.457
E/E'	7.19±1.46	7.24±1.19	0.160	0.873
LVEF (%)	67.37±3.64	67.19±3.74	0.204	0.839

A=left atrioventricular valve late diastolic blood flow peak; CMD=coronary microvascular dysfunction; E=left atrioventricular valve early diastolic blood flow peak; E'=left atrioventricular annulus diastolic early motion velocity; LVEDd=left ventricular end-diastolic diameter; LVEF=left ventricular ejection fraction; LVESd=left ventricular end-systolic diameter

Comparison of Left Ventricular Strain Parameters Between the Groups

A comparison of GRS and GCS showed no significant difference between the groups (*P*>0.05). GLS of the CMD group was lower than that of the control group (-17.63±1.50 *vs*. -20.09±1.54, respectively), and GAS of the CMD group was lower than that of the control group (-29.68±1.60 *vs*. -32.78±2.06, respectively), presenting a highly significant difference(*P*<0.001) (Table 3).

**Table 3:** Comparison of left ventricular strain parameters between the groups.

	Control group	CMD group	t	*P*-value
n	30	42		
GLS (%)	-20.09±1.54	-17.63±1.50	-6.756	0.000
GRS (%)	44.35±8.57	44.77±5.97	-0.247	0.806
GCS (%)	-23.38±2.73	-22.46±1.63	-1.776	0.080
GAS (%)	-32.78±2.06	-29.68±1.60	-1.786	0.000

CMD=coronary microvascular dysfunction; GAS=global area strain; GCS=global circumferential strain; GLS=global longitudinal strain; GRS=global radial strain

Comparison of Conventional Echocardiography Parameters and Left Ventricular Strain Parameters Between Male and Female Subjects

As shown in Table 4, there is no statistical difference in each conventional echocardiography parameter or each left ventricular strain parameter between male and female subjects in the control and CMD groups (*P*>0.05).

**Table 4:** Comparison of conventional echocardiography parameters and left ventricular strain parameters between male and female subjects in the groups.

Parameter	Control group		t	*P*-value	CMD group		t	*P*-value
	Male	Female			Male	Female		
n	15	15			23	19		
LVEDd (mm)	45.88±2.82	44.04±3.80	1.512	0.142	45.82±1.85	45.91±2.59	-0.122	0.904
LVESd (mm)	26.34±3.01	25.29±2.74	1.005	0.324	25.60±2.96	26.62±3.44	-1.033	0.308
E/A	1.35±0.19	1.47±0.15	-1.908	0.067	1.42±0.21	1.31±0.28	1.482	0.146
E/E'	6.95±1.05	7.43±1.78	-0.898	0.377	7.23±1.25	7.25±1.16	-0.059	0.953
LVEF (%)	66.67±4.51	68.07±2.46	-1.054	0.301	66.61±3.81	67.89±3.63	-1.111	0.273
GLS (%)	-20.14±1.63	-20.04±1.51	-0.167	0.868	-17.83±1.12	-17.40±1.88	-0.924	0.361
GRS (%)	45.18±7.75	43.51±9.52	0.530	0.600	44.46±6.23	45.14±5.79	-0.359	0.721
GCS (%)	-23.72±3.05	-23.03±2.43	-0.689	0.496	-22.62±1.74	-22.26±1.50	-0.717	0.478
GAS (%)	-32.77±2.01	-32.78±2.17	0.020	0.984	-29.38±1.72	-30.04±1.41	1.348	0.185

A=left atrioventricular valve late diastolic blood flow peak; CMD=coronary microvascular dysfunction; E=left atrioventricular valve early diastolic blood flow peak; E'=left atrioventricular annulus diastolic early motion velocity; GAS=global area strain; GCS=global circumferential strain; GLS=global longitudinal strain; GRS=global radial strain; LVEF=left ventricular ejection fraction; LVEDd=left ventricular end-diastolic diameter; LVESd=left ventricular end-systolic diameter

### Univariate Logistic Regression Analysis

For univariate logistic regression analysis, the occurrence of CMD was used as the dependent variable (assignment: CMD=1, control=0), and GLS, GRS, GCS, and GAS were included as independent variables. The results showed that GLS and GAS were the influencing factors of CMD (Table 5).

**Table 5:** Univariate logistic regression analysis of the factors possibly associated with CMD.

Variable	Β	SE	Wald	Df	*P*-value	OR	95%CI
GLS	1.262	0.392	10.363	1	0.001	3.534	(1.638, 7.621)
GRS	0.061	0.058	1.105	1	0.293	1.063	(0.949, 1.190)
GCS	0.370	0.210	3.110	1	0.078	1.447	(0.960, 2.182)
GAS	0.862	0.251	11.805	1	0.001	2.368	(1.448, 3.872)
Constant	56.675	14.030	16.319	1	0.000	4,00E+24	

B=regression coefficient; CI=confidence interval; CMD=coronary microvascular dysfunction; Df=degree of freedom; GAS=global area strain; GCS=global circumferential strain; GLS=global longitudinal strain; GRS=global radial strain; OR=odds ratio; SE=standard error

### Receiver Operating Characteristic Curve

**Fig. 2:**
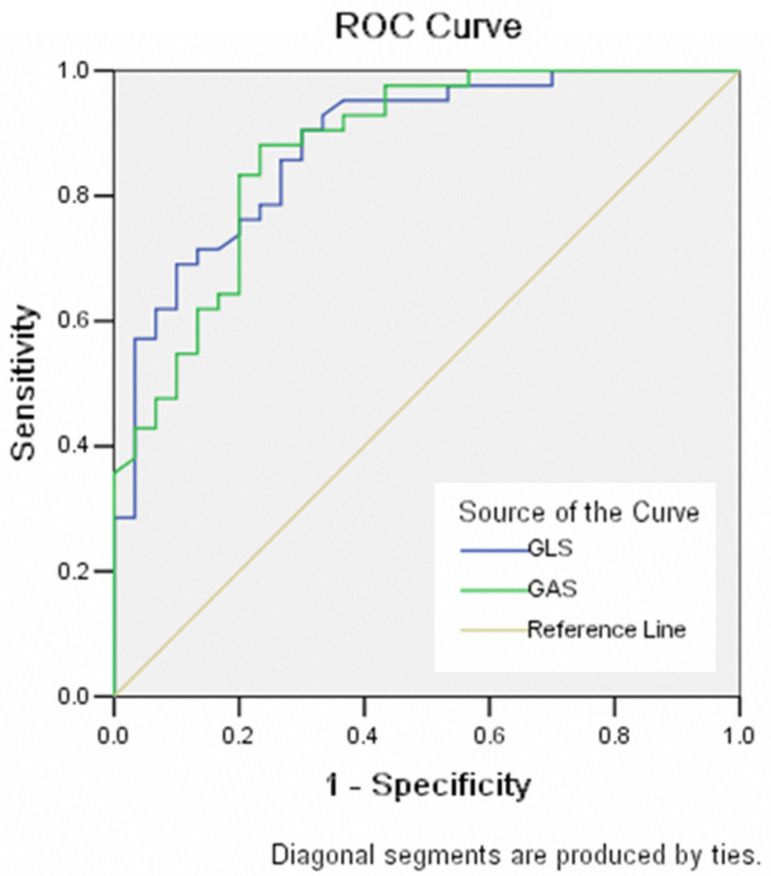
Receiver operating characteristic (ROC) curve of global longitudinal strain (GLS) and global area strain (GAS) markers for diagnosis of patients with coronary microvascular dysfunction.

The area under the ROC curve for use of GLS to diagnose CMD was 0.883, and the area under the ROC curve for use of GAS to diagnose CMD was 0.875. Both parameters had a high diagnostic sensitivity and specificity (Figure 2).

### Prognosis Analysis

**Fig. 3:**
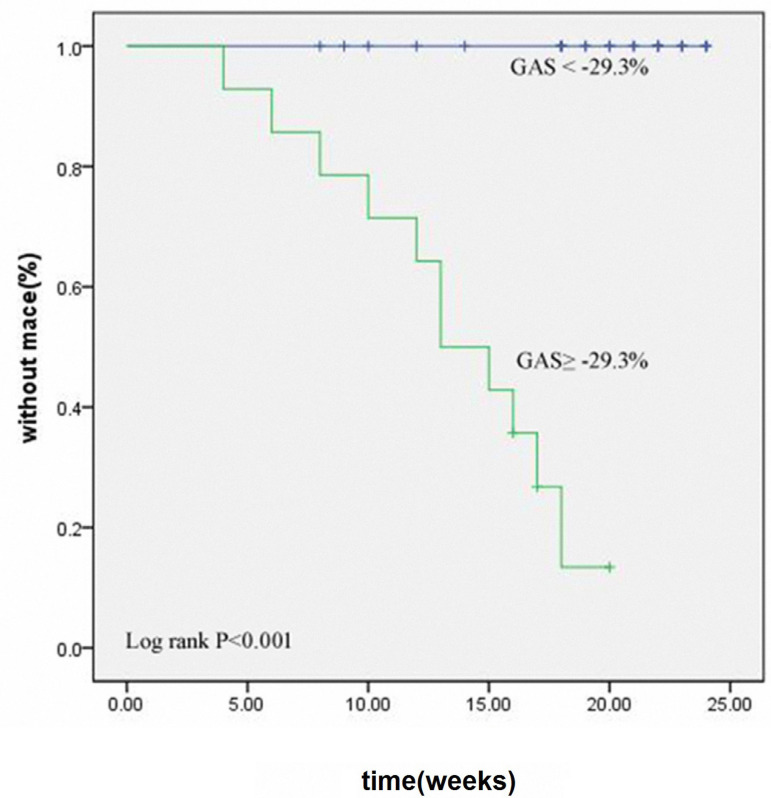
Survival analysis according to global area strain (GAS) markers. There were significant differences between the GAS < -29.3% group and the GAS ≥ -29.3% group. MACE=major adverse cardiac events

For patients in the CMD group, a significant difference was found in GAS (t=6.03, *P*<0.001) by comparing the strain parameters according to the occurrence of MACE during the 24-week follow-up. A total of 11 of 42 CMD patients developed MACE, accounting for 26.2% of the group. Based on the abovementioned indexes, the ROC curve showed that the area under the curve of GAS was 0.950. According to the evaluation method of best diagnostic threshold, the maximum Youden’s index was calculated to be 0.839, and the corresponding main cutoff value was -29.3% (sensitivity, 100%; specificity, 83.9%). Kaplan-Meier survival curve analysis was conducted after grouping according to this cutoff value. It indicated that a GAS of -29.3% (log-rank test chi-square=34.245, *P*<0.001) was a strong predictor of MACE (Figure 3).

## DISCUSSION

CMD is caused by the abnormal regulation of coronary artery systolic function, including a decreased blood supply of coronary microvessels and impaired endothelial function of microvessels. The coronary microvasculature (which provides the vascular resistance), nutrient supply of cardiac cells, and elimination of metabolic products can directly affect the perfusion of downstream cardiac cells ^[4]^. The prevalence of CMD may depend on the degree of endothelial and microvascular dysfunction in patients, and a high proportion of CMD patients have abnormal coronary blood flow ^[4]^. At present, the increasing attention is being paid on the diagnosis and treatment of CMD. Currently, most CMD patients have a good prognosis, and the incidence of cardiovascular events is the same as that of the normal population. However, 20-30% of CMD patients have gradually aggravating angina symptoms that greatly affect the quality of life ^[5]^. Some challenges still exist in the treatment of patients without structural coronary artery disease, and current management strategies are focused on symptomatic remission ^[6]^. Therefore, it is very important to seek early diagnostic indicators for disease evaluation and prognosis. There is increasing interest in the measurement of globe myocardial strain because it is a sensitive and robust index to detect subclinical myocardial dysfunction ^[7]^. This study evaluated the diagnostic value of strain indexes observed by 3D-STI technique for CMD.

Traditional two-dimensional ultrasonic technique, due to its single-plane detection, is not an effective evaluation method. In particular, the sensitivity and specificity of this method are not satisfactory for the measurement of cardiac function changes due to a 10% reduction of the LVEF. Conflicting results have been reported in earlier studies using cardiovascular magnetic resonance imaging perfusion to detect myocardial ischaemia as a marker of CMD ^[8]^. Because the examination time is very long, some patients cannot tolerate this method. Therefore, its clinical application is not extensive. 3D-STI technique can be used to evaluate the strain in different directions of the left ventricle from a three-dimensional perspective, overcoming the drawbacks of two-dimensional speckle-tracking imaging technique. Furthermore, 3D-STI technique provides more abundant information for the evaluation of subclinical myocardial injury of the left ventricle ^[9]^. The global strain and systolic function of the left ventricle are directly quantified by measuring the peak strain in the radial, longitudinal, and circumferential directions ^[10]^. Due to the lack of angle dependence, 3D-STI technique can accurately distinguish the position of myocardial spots and assess myocardial function by local and global strain ^[11]^. This method has been widely used in clinical and experimental studies to evaluate the overall and local myocardial function of the left ventricle. However, few studies have been reported its application for CMD.

This study showed that the longitudinal strain of patients in the CMD group was significantly decreased. Due to poor hypoxia tolerance of the endocardium, if the coronary artery blood supply is decreased, the endocardial myocardium will be involved due to the longitudinal strain, which is mainly responsible for the significant changes observed. We also found that the area strain of the CMD group was decreased, resulting in an obvious diagnostic and prognostic value. The area strain is mainly determined by combining the longitudinal strain with the circumferential strain in 3D-STI. It refers to the size of the area of deformation between the inner and outer membranes of the heart, which comprehensively reflects the movement of the ventricular muscle in all directions ^[12]^. Additionally, the area strain is a three-dimensional comprehensive index that affects the area of deformation of the middle myocardium during the systole and diastole ^[9]^. Related research suggests that small changes in left ventricular function can be reflected by the area strain. Although the decreases in the global, longitudinal, and circumferential systolic peak strain are not obvious, the area strain may show statistically significant changes ^[13]^. The longitudinal strain changed, but the study found that the difference was not obvious. Therefore, the superiority of the area strain was demonstrated. Moreover, the area strain shows significant advantages in predicting MACE. We found that the area strain played an extremely important role in predicting the clinical prognosis according to the Kaplan-Meier survival curve analysis, which was an innovation of this study.

### Limitations

This study has some limitations. The sample size of this study is relatively small. A larger sample size will make the results more convincing. In our next studies, the sample size should be further increased for obtaining more satisfactory outcomes.

## CONCLUSION

In summary, for CMD patients, the conventional ultrasound cannot detect early left ventricular heart abnormalities, while 3D-STI technique has obvious advantages. According to the analysis, GLS and GAS of the CMD patients are significantly lower than those of the control group. Further use of GLS and GAS values can improve the sensitivity and specificity of diagnosis and facilitate the early diagnosis and assessment. GAS can be used to assess the survival prognosis early at a cutoff point of -29.3%, which is of great significance for clinical practice. However, in view of the small sample size at present, we will continue to expand the sample size to verify the reliability of the index.

**Table:** 

Abbreviations, acronyms & symbols				
3D-STI	= Three-dimensional speckle-tracking imaging		GAS	= Global area strain
A	= Left atrioventricular valve late diastolic blood flow peak		GCS	= Global circumferential strain
B	=Regression coefficient		GLS	= Global longitudinal strain
CI	= Confidence interval		GRS	= Global radial strain
CMD	= Coronary microvascular dysfunction		LVEDd	= Left ventricular end-diastolic diameter
CTA	= Computed tomography angiography		LVEF	= Left ventricular ejection fraction
Df	= Degree of freedom		LVESd	= Left ventricular end-systolic diameter
E	= Left atrioventricular valve early diastolic blood flow peak		MACE	= Major adverse cardiac events
E '	= Left atrioventricular annulus diastolic early motion velocity		OR	= Odds ratio
ED	= End-diastolic		ROC	= Receiver operating characteristic
ES	= End-systolic		SE	= Standard error
